# Defining and improving quality management in Dutch diabetes care groups and outpatient clinics: design of the study

**DOI:** 10.1186/1472-6963-13-129

**Published:** 2013-04-05

**Authors:** Marjo JE Campmans-Kuijpers, Lidwien C Lemmens, Caroline A Baan, Kees J Gorter, Jolanda Groothuis, Klementine H van Vuure, Guy EHM Rutten

**Affiliations:** 1 Julius Center for Health Sciences and Primary Care, University Medical Centre Utrecht, Heidelberglaan 100, 3508, GA Utrecht, The Netherlands; 2 Centre for Nutrition, Prevention and Health Services, National Institute of Public Health and the Environment, A. van Leeuwenhoeklaan 9, 3721, MA Bilthoven, The Netherlands; 3 Knowledge Centre for Shared Care, Dr Spanjaardsweg 11, 8000, GK Zwolle, The Netherlands

**Keywords:** Type 2 diabetes, Quality management, Quality improvement, Care groups, Outpatient clinics, Questionnaire, Integrated care, Shared care, Disease management

## Abstract

**Background:**

Worldwide, the organisation of diabetes care is changing. As a result general practices and diabetes teams in hospitals are becoming part of new organisations in which multidisciplinary care programs are implemented. In the Netherlands, 97 diabetes care groups and 104 outpatient clinics are working with a diabetes care program. Both types of organisations aim to improve the quality of diabetes care. Therefore, it is essential to understand the comprehensive elements needed for optimal quality management at organisational level. This study aims to assess the current level of diabetes quality management in both care groups and outpatient clinics and its improvement after providing feedback on their quality management system and tailored support.

**Methods/design:**

This study is a before-after study with a one-year follow-up comparing the levels of quality management before and after an intervention to improve diabetes quality management. To assess the status of quality management, online questionnaires were developed based on current literature. They consist of six domains: organisation of care, multidisciplinary teamwork, patient centeredness, performance management, quality improvement policy and management strategies. Based on the questionnaires, respondents will receive feedback on their score in a radar diagram and an elucidating table. They will also be granted access to an online toolbox with instruments that proved to be effective in quality of care improvement and with practical examples. If requested, personal support in implementing these tools will be available. After one year quality management will be measured again using the same questionnaire.

**Discussion:**

This study will reveal a nationwide picture of quality management in diabetes care groups and outpatient clinics in the Netherlands and evaluate the effect of offering tailored support. The operationalisation of quality management on organisational level may be of interest for other countries as well.

## Background

Worldwide, the number of type 2 diabetes mellitus patients (T2DM) is rising rapidly [1]. This poses great challenges to cost, efficacy and quality of diabetes care. Diabetes care usually involves many health care providers. Consequently, optimal collaboration and coordination among professionals has become essential for delivering high quality of care [2]; in addition, this care should be organised in a patient centred way [3]. Organisations providing diabetes care are obliged to control these complex processes by quality management (QM). QM comprises procedures to monitor, assess and improve the quality of care [4]. Besides focussing on patient related and process outcomes, also other aspects of QM on an organisational level are likely to become crucial in order to maintain or enhance the delivery of good quality diabetes care [5].

In the Netherlands, patients with type 2 diabetes are in principle treated in primary care. Patients, who need more complex care, are treated in secondary care [6]. In primary care, most patients (80-85%) are treated within so called diabetes care groups (DCGs) [7]. These DCGs, comparable with Accountable Care Organizations (ACOs) [8,9] in the United States and Clinical Commission Groups (CCGs) [10] in the United Kingdom, emerged after the introduction of bundled payment in 2007 [11]. DCGs are the main contractor of a diabetes care program, and are responsible for the organisation, coordination and delivery of diabetes care. They comprise between three and 250 general practitioners (GPs) [12]. The diabetes care program is based on the Dutch Diabetes Federation Health Care Standard (DFHCS) for T2DM [13]. Patients who need more complex diabetes care are treated in 104 diabetes outpatient clinics (DOCs). Besides the DFHCS standard, the latter have special guidelines for treatment of a diabetic foot, retinopathy, nephropathy [6,14].

The organisation of DCGs [15] and DOCs [16] varies widely. In DOCs the organisation is managed by the hospital department itself, although the endocrinologist is mainly responsible for the quality of diabetes care. In the DCGs, managers, managing directors or GPs are in charge of a whole care group; their type of organisation varies with regard to the type of legal entity, the ownership of the care group and the number of employees [15]. Also the number of patients treated in both DCGs (400–22,500) [12] and DOCs (250–4,500) varies widely. Both DCGs and DOCs strive to deliver good quality of care for type 2 diabetes patients. Very recently, Tricco et al. (2012) stated that targeting the system in chronic care management is important in improving diabetes care [17]. So far, little attention has been paid to QM on an organisational level. Therefore, this study focuses on improving quality of diabetes care at an organisational level by improving QM.

First, we want to study the current level of QM in all DCGs and DOCs in the Netherlands. Based on the baseline measurement participating organisations will be given feedback; this will show them their strengths and weaknesses in QM. Next, they will be provided access to a toolbox for QM and offered the possibility of tailored support. After one year we will examine their level of QM again.

In this paper we describe the study design and the operationalisation of QM and the questionnaire used to measure QM.

## Methods/design

### Study design

This study is a before-after study with a one-year follow-up comparing the levels of QM before and after an intervention to improve QM within DCGs and DOCs. The study was funded by the Dutch Diabetes Federation (grant no NAD 3.05) and no ethical approval was needed because the study does not meet the WMO (Wet Medisch wetenschappelijk Onderzoek) criteria for medical human scientific research [18].

### Defining quality of care and quality management

There are many definitions of quality of care depending on the perspective used. Patients mainly focus on effectiveness and access of care, and they expect consistent information. Care providers and insurers want to deliver effective care according to the latest standards appropriate for their patients. For care managers and insurers efficiency and safety of care are main issues. Hence, good quality of care can be defined as ‘care of a high standard that is effective, efficient, safe and patient oriented’ [19].

Quality of care is not merely a random outcome, but at least partially the outcome of a controlled process: quality management. Thus, QM can be defined as “all procedures explicitly designed to monitor, assess, and improve the quality of care” [4].

#### Operationalising and measuring QM

To measure the level of QM, a suitable questionnaire was needed. Therefore, we first formulated criteria for measuring diabetes QM. A suitable questionnaire should target the organisational level of health care organisations and focus on diabetes care.

We performed an extensive literature search for suitable questionnaires in the MEDLINE®, EMBASE®, CINAHL®, and Cochrane databases and in the Cochrane registry for terms relevant to diabetes type 2 and outpatient clinics, ambulatory care, managed care, shared care, integrated care, coordinated care, Accountable Care Organizations, Health Maintenance Organization, disease management, and quality improvement, total quality management, continuous quality improvement or Plan-Do-Check-Act cycle. For the exact search terms see Additional file 1.

**Figure 1:**
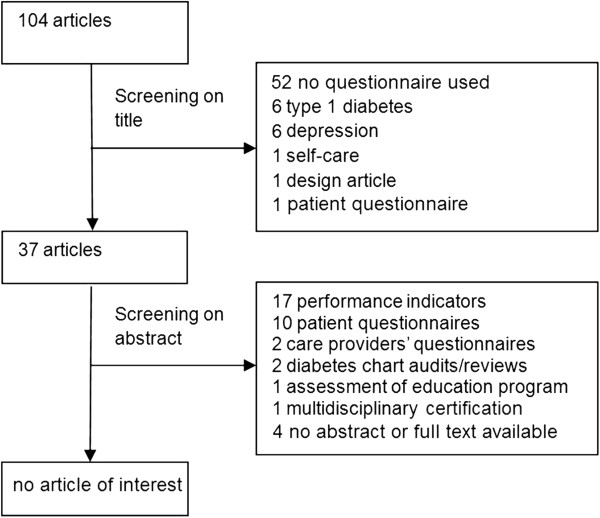
Flowchart of search for questionnaires suitable for measuring diabetes QM on organisational level.

No questionnaires measuring QM at an organisational level were found in medical literature (Figure 1). Consequently, we had to develop a questionnaire ourselves; therefore we first needed to itemize the domains we considered crucial in QM. Hence, we studied health care models, including those based on industrial models, to see how QM was operationalised. We found seven models containing different domains of QM: the Chronic Care Model (CCM) [20]; the ‘Harmonisatie Kwaliteitsbeoordeling in de Zorgsector’ (HKZ: Harmonisation Quality Assessment in Health Care) model, based on ISO 9001 [21]; The Malcolm Baldrige USA National Quality Award (MBQA) [22]; The European Foundation for Quality Management (EFQM) [23]; the ‘Instituut Nederlandse Kwaliteit’ (INK: Institute for Dutch Quality) model from the Dutch Governmental foundation, based on the above mentioned EFQM [24,25]; and a measuring instrument developed by C. Wagner, which was later adapted in the Quality and Safety Management in Hospitals (QSMH) questionnaire [26,27]; and the Developmental Model for Integrated Care (DMIC) [28].

Comparing these seven models (Table 1), nine common domains of QM emerged. Here we describe these common domains, albeit sometimes with different labels.

1. **Organisation of care:** The CCM mentions ‘delivery system design’. According to the CCM ‘Clinical information systems’ are necessary to be able to build an effective ‘delivery system design’ and are therefore considered part of the organisation of care. Organisation of care is further reflected in DMIC by ‘delivery system’, in MBQA by ‘management of process quality’, in EFQM by ‘processes’ and in INK by ‘management of processes’, QSMH mentions ‘process control based on standards’ and ‘quality assurance documents’. Hence, we will regard organisation of care as a separate domain.

2. **Multidisciplinary teamwork:** DMIC mentions ‘inter-professional teamwork’ and HKZ ‘care chain quality’. In the CCM multidisciplinary teamwork is not described as a domain, however, the CCM explicitly aims to create a productive interaction between an informed, activated patient and a prepared, proactive practice team. In MBQA, EFQM, INK and QSMH models multidisciplinary teamwork is not a separate domain. However, ‘partnership & resources’ of EFQM can be interpreted as multidisciplinary teamwork. As multidisciplinary teamwork is essential in organising diabetes care, we will consider it a separate domain.

3. **Patient centeredness** is a special domain in the DMIC; HKZ lists ‘focus on client perspective’. In CCM patient centeredness is reflected in ‘self-management support’, QSMH measures ‘involvement of patients’, and MBQA lists ‘customer focus and satisfaction’. Patients are mentioned as ‘clients and partners’ by INK and as ‘customer results’ by EFQM. As diabetes care should be organised patient centred [3], patient centeredness will be a special domain.

4. **Performance management:** is mentioned in DMIC. In MBQA performance results is reflected in ‘information and analysis’. EFQM has four boxes for results; within these boxes performance management is reflected by ‘key performance results’. INK lists four result dimensions; but they do not explicitly mention performance management. In HKZ results are part of the domain ‘improvement’. CCM focuses more on the care process, less on results, although ‘decision support’ and ‘clinical information systems’ both support performance management. QSMH includes ‘process improvement based on QI procedures’. Performance management will be a domain in our questionnaire. At organisational level we will be mainly interested in which results are measured in what way and how these results are handled.

5. **Quality improvement (QI) policy:** is mentioned by MBQA as ‘quality and operational results’ and by HKZ as ‘improvement’ and ‘client safety’. In DMIC ‘result-focused learning’ and ‘quality care’ together cover QI. Both EFQM and INK define QI as the main purpose of the model. EFQM and INK both do mention ‘strategy and policy’. QSMH mentions ‘process improvement based on QI procedures’. CCM mentions ‘decision support’ to improve the knowledge and skills of providers; this can be seen as part of QI policy. On the other hand, the aim of the whole CCM is to guide QI in chronic care. Since QI is likely to be one of the major aims of each QM organisation, it will be a separate domain in our tool.

6. **Management strategies** and leadership are reflected in the followings domains of the models: ‘health care organization’ (CCM), ‘professional behaviour’ and ‘ISO-compatibility’ (HKZ), ‘leadership’ (MBQA, EFQM, INK). MBQA also mentions ‘strategic quality planning; INK mentions ‘management and financiers’ and ‘management of resources’. Leadership is important in the QSMH model too, but it states it is difficult being measured without bias [26]; therefore they concentrate on ‘quality assurance documents’. DMIC divides leadership into three clusters: ‘roles and tasks’, ‘commitment’ and ‘transparent entrepreneurship’. Since management is responsible for the mission and coordination of care, it will be a separate domain in our questionnaire, taking bias on questioning the manager into account. Further, to reduce this bias, the term leadership will be left out in the name of this domain.

7. **Personnel** are considered a separate domain in the MBQA reflected by ‘human resource development and management’; in EFQM by ‘people’ and ‘people results’; in INK by ‘management of employees’ and ‘personnel’. QSMH is focussed on hospitals and mentions ‘human resources management’. CCM does not have a domain for personnel. HKZ does not mention personnel. We will not regard personnel as a separate domain because care groups are very differently organised; in some of them the central organisation only consists of one director. In hospitals the organisation consists of more personnel. However, the training of diabetes care providers remains an important issue; it will be covered in the domain ‘quality improvement policy’.

8. **Safety** is only mentioned by HKZ. Though it is an important part of quality and QM as well, we think it could be covered by ‘quality improvement policy’.

9. **Community** is mentioned as ‘community resources’ in CCM and ‘community’ in INK and ‘society results in ‘EFQM’. Because our focus is on the organisation itself, we will not regard community as a special domain.

**Table 1:** Different domains across different quality management systems

**CCM**	**HKZ**	**MBQA**	**EFQM**	**INK**	**QSMH**	**DMIC**
A framework to guide QI in chronic care. CCM aims to create a productive interaction between an informed, activated patient and a prepared, proactive practice team.	The aim of HKZ is harmonisation of quality assessment in healthcare and wellbeing.	To improve competitiveness in healthcare QI by creating awareness of the importance of QI, recognition of accomplishments and information transfer	A European model which supports organisations to self-assess and reflect its level of organization in order to improve its organization.	A model based on EFQM to support profit and non-profit organizations to get to excellent achievements	A measuring instrument for evaluation of quality systems	A quality management model developed for integrated care.
	HKZ is based on ISO 9001					
• community resources	• improvement	• leadership	**5 boxes for enablers:**	**5 organisational dimensions:**	**5 focal areas for QI activities:**	**9 clusters:**
• health care organisation	• focus on client perspective	• Information and analysis	• leadership	• leadership	• quality assurance documents	• patient-centeredness
• self-management support	• client safety	• strategic quality planning	• people	• strategy and policy	• involvement of patients	• delivery system
• decision support	• professional behaviour	• human resource development and management	• policy& strategy	• management of employees	• process control based on standards	• performance management
• delivery system design	• care chain quality	• management of process quality	• partnership& resources	• management of resources	• human resources management	• quality care
• clinical information system	• ISO-compatibility	• quality and operational results	• processes	• management of processes	• process improvement based on QI procedures	• result-focused learning
		• customer focus and satisfaction	**4 boxes for results:**	**4 result dimensions:**		• interprofessional teamwork
			• people results	• clients and partners		• roles and tasks
			• customer results	• personnel		• commitment
			• society results	• community		• transparent entrepreneurship
			• key performance results	• management and financiers		

CCM: Chronic Care Model; HKZ: Harmonisatie Kwaliteitsbeoordeling in de Zorgsector (Harmonisation Quality Assessment in Health Care); MBQA: The Malcolm Baldrige USA National Quality Award; EFQM: The European Foundation for Quality Management; INK: Instituut Nederlandse Kwaliteit (Institute for Dutch Quality); QSMH: Quality and Safety Management in Hospitals; DMIC: The Developmental Model for Integrated Care; QI: Quality improvement.

After thorough discussions between the authors about the different domains of QM, consensus was reached to distinguish six domains within diabetes care QM, namely: organisation of care, multidisciplinary teamwork, patient centeredness, performance management, quality improvement policy and management strategies. These domains were then operationalised into sub-domains, to be more specific about the content/definition of the domains. Based on the above mentioned models we defined a total of 28 sub-domains, which are shown in Table 2.

**Table 2:** Results of the weighing of the importance of sub-domains within domains by two expert panels

**(Sub) Domains**	**Care groups (DCGs)**				**Outpatient clinics (DOCs)**			
	**Number of questions per sub-domain**	**Mean** **(%)**	**Median (min,max) (%)**	p-value*	**Number of questions per sub-domain**	**Mean** **(%)**	**Median (min,max) (%)**	p-value*
**Organisation of care**	**13:**	**100.0**			**9:**	**100.0**		
Care program	4	**34.6**	33.3(25–50)	ns	4	**32.0**	33.3(20–50)	ns
Continuity and Coordination	6	**31.5**	30(25–50)	ns	2	**31.5**	33.3(20–40)	ns
Communication and Information	3	**34.0**	33.2(20–50)	ns	3	**36.5**	33.3(25–60)	ns
**Multidisciplinary teamwork:**	**15:**	**100.0**			**16:**	**100.0**		
Work agreement	4	**24.0**	25(11–33)	ns	3	**23.0**	20(10–50)	ns
Tasks and responsibilities	3	**25.4**	25(17–35)	ns	3	**23.5**	20(10–50)	ns
Teamwork/ consultation/ shared education/ guidelines	5	**26.0**	25(16–34)	ns	6	**16.3**	16.7(10–25)	ns
Transfer and referral	3	**24.6**	25(13–42)	ns	3	**17.8**	20(10–25)	ns
Diabetic foot team	-	**-**	-	-	1	**19.4**	20(5–30)	ns
**Patient centeredness:**	**7:**	**100.0**			**7:**	**100.0**		
Self-management	1	**20.2**	20(5–35)	ns	1	**31.1**	30(5–60)	0.04
Individual care plan	1	**18.7**	18.3(10–30)	ns	1	**12.3**	15(5–20)	0.03
Policy on patient education	1	**12.2**	12.5(5–20)	0.02	1	**17.3**	16(5–30)	ns
Inspection of medical file	2	**13.7**	15(5–20)	ns	2	**11.8**	10(5–16)	0.00
Patient interests	1	**16.2**	15(10–25)	ns	1	**12.9**	10(5–20)	ns
Patient involvement	1	**19.2**	15.8(10–40)	ns	1	**14.6**	10(10–40)	ns
**Performance management:**	**8:**	**100.0**			**8:**	**100.0**		
Registering results	1	**28.0**	25(10–40)	ns	1	**30.6**	30(10–55)	0.04
Control of results	1	**18.5**	20(5–30)	ns	1	**20.6**	20(10–40)	ns
Processing of results	2	**16.5**	17.5(5–40)	ns	2	**10.6**	10(5–20)	0.00
Analysing results	2	**16.0**	17.5(5–30)	ns	2	**17.8**	15(10–50)	ns
Performance indicators	2	**21.0**	20(10–40)	ns	2	**20.6**	20(10–30)	ns
**Quality improvement:**	**11:**	**100.0**			**11:**	**100.0**		
Elements of QI	1	**12.5**	10(0–30)	ns	1	**12.8**	15(0–20)	ns
Feedback/ benchmark	2	**25.5**	27.5(15–30)	0.00	2	**17.2**	20(0–30)	ns
Visitation	2	**19.5**	20(0–40)	ns	1	**21.1**	20(10–35)	ns
Education	2	**21.0**	20(10–35)	ns	2	**15.0**	20(0–20)	ns
Patient safety	2	**9.5**	10(0–20)	0.01	3	**18.3**	15(10–35)	ns
Defining sub-groups	2	**12.0**	12.5(0–25)	ns	2	**15.6**	15(0–30)	ns
**Management strategies:**	**5:**	**100.0**			**6:**	**100.0**		
Structural policy	3	**54.7**	50(33–100)	0.02	3	**47.9**	40(33–100)	ns
Quality system	1	**16.3**	20(0–33)	0.01	1	**22.3**	27.5(0–40)	0.03
Quality documents	1	**29.0**	33(0–40)	ns	2	**29.8**	31.7(0–50)	ns

*Significant difference versus equally weighting: ns = not significant.

#### Questionnaire

Based on the domains and sub-domains, two separate, yet comparable questionnaires for DCGs and for DOCs were developed to be able to adjust to different organisational backgrounds in DCGs and DOCs. In a pilot study, both draft questionnaires were tested by four and five experts from primary and secondary care respectively. For each sub-domain one to six questions were included in the final questionnaires; the total number of questions amounts to 51 for DCGs and 48 for DOCs. In the DCG questionnaire, the diabetic foot team was left out; and the DOC questionnaire contained a looping to prevent posing irrelevant questions.

#### Weighing of sub-domains

Theoretically, all questions can be weighted equally with one point (thus giving each sub-domain equal importance within a domain) or the questions can be weighted by an expert panel [29]. We asked two expert panels to weigh the sub-domains: the DCG expert panel consisted of seven managers, one staff member, one quality manager and one diabetes nurse; a DOC expert panel consisted of two managers, three endocrinologists and four diabetes nurses. There were significant differences (one-sample t-tests) between equal weighting of each sub-domain and the weight given by the expert panels. Therefore, each question will be given one point; all questions together within a sub-domain contribute X% to the maximum score of a domain, where X is the mean weight given by the corresponding expert panel (Table 2). For each organisation the score in the domains and sub-domains will be computed. The total score on QM is the mean score of all domains.

#### Agreement and validity of questionnaire

To test the validity of the questionnaire the percentage of agreement will be checked by inviting two persons of the same organisations to fill out the questionnaire.

### Improving QM

#### Study population and recruitment

All DCGs (n = 97) and DOCs (N = 104) will be invited to fill out the online questionnaires. Two persons per organisations will be given the opportunity to do so. Personal email-addresses will be collected for this invitation, to be able to send participants the results personally. This policy will offer participants the opportunity to determine with whom they want to share their results. After two and four weeks reminders will be sent. After six weeks people who partly completed the questionnaire will be emailed and phoned to complete their questionnaire.

#### Intervention

The intervention consists of 1. giving organisations feedback on their current level of diabetes QM based on the scores of the questionnaire; 2. providing access to a toolbox for QM; and 3. offering the possibility of tailored support.

##### Feedback

**Figure 2:**
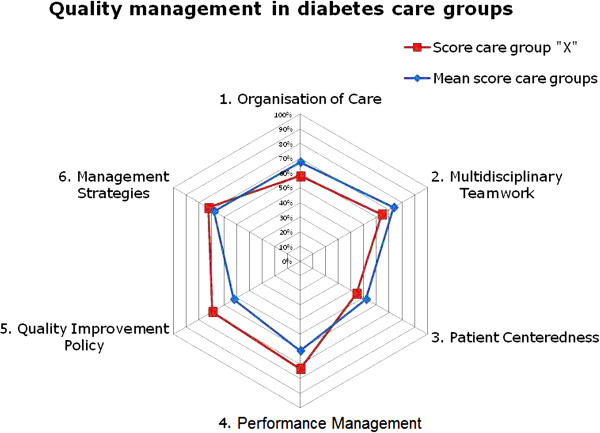
An example of feedback to a care group.

Within one month after responding on the online questionnaire, the responders will be given feedback on the level of the diabetes QM in their organisation. To be able to quickly discern their strengths and weaknesses in QM, their results will be presented in a radar diagram consisting of the six QM domains (Figure 2) and in a table elucidating the scores of the domains and sub-domains; both results will be compared to the results of the group of DCGs / DOCs as a whole (Table 3).

**Table 3:** An example of feedback to a care group

**Domains**	**Sub domains**	**Your organisation [%]**	**Mean score care groups [%]**
1 Organisation of Care			
	Care program	**90**	81
	Continuity and coordination	**60**	72
	Communication en information	**21**	49
^*^Mean weighted score organisation of care:		**58**	67
2 Multidisciplinary teamwork			
	Work agreement	**61**	60
	Tasks and responsibilities	**78**	92
	Teamwork/consultation /shared education /guidelines	**45**	74
	Transfer and referral	**75**	68
^*^Mean weighted score multidisciplinary teamwork:		**64**	74
3 Patient centeredness			
	Self-management	**25**	94
	Individual care plan	**33**	30
	Policy on patient education	**50**	58
	Inspection of medical file	**17**	39
	Patient interests	**71**	60
	Patient involvement	**67**	14
^*^Mean weighted score patient centeredness:		**44**	49
4 Performance management			
	Registering results	**63**	60
	Control of results	**40**	27
	Processing of results	**100**	67
	Analysing results	**68**	56
	Performance indicators	**100**	94
^*^Mean weighted score performance results:		**73**	61
5 Quality improvement policy			
	Elements of quality improvement	**60**	38
	Feedback/ benchmark	**100**	73
	Visitation	**60**	43
	Education	**100**	67
	Patient safety	**0**	22
	Defining sub-groups	**33**	53
^*^Mean weighted score quality improvement policy:		**69**	54
6 Management Strategies			
	Structural policy	**92**	66
	Quality system	**67**	56
	Quality documents	**40**	72
^*^mean weighted score on management strategies:		**73**	66
**mean total score quality management**		**64**	**62**

*The weight of a sub-domain within a domain is determined by consulting experts.

##### Access to online toolbox

Together with receiving feedback, all participants will receive an e-mail letter elucidating the feedback and granting them access to the online toolbox. In this toolbox we gathered instruments for QI and practical examples of good practices in diabetes care per domain. Participating DCGs and DOCs will also be given the opportunity to share tools with other organisations. These tools should clearly been described and tested and proven effective or they have been developed in an evidence based manner or they are proven effective in an interventional study. The use of the toolbox will be monitored during the one-year follow-up period, to quantify its use.

##### Tailored support on request

Within a three months period after the feedback with the radar diagram, all respondents will be telephonically asked about whether they studied the feedback, looked into the toolbox or discussed the results within their organisation and whether they need support in improving their QM. If so, tailored support can be provided in three different ways: firstly, more information on the study or the results of the questionnaire, secondly, advice by phone or thirdly a visit by two experienced quality consultants. In these latter two ways, these consultants together with the participating organisation analyse which gaps in QM could be tackled first. The consultants can give suggestions, offer supportive tools or show the participants how to initiate a QI strategy; for this purpose they have dedicated time up to a maximum of ten hours per organisation. The kind of support that will be given and the time spent will be monitored.

### Outcome measures

The scores in the six domains and 28 sub-domains will be calculated using the mean weight given by the corresponding expert panel. The overall score in QM of an organisation is the mean score of the six domains. The overall score of the whole group will reflect the level of QM in DCGs and DOCs.

After one year, we will measure the level of QM again and in the same way. The change in QM will be computed as the difference in the overall score in QM at the start of the study and after one year. Further, the one-year change of the separate domains will be computed, to assess which domain changed the most.

For measurements concerning the intervention, the quality consultants will register the time spent on the support of each organisation. After one year, the participating organisations will be asked whether the first feedback triggered them to adapt parts of their QM policy and if yes; which parts. Further, they will be asked whether they used the toolbox and how much time (hours, rough estimation) they spent extra on QM during the intervention.

To assess the generalisability of the study results and check whether selection bias occurred, organisations who did not participate will be asked how many diabetes patients are being treated by their organisation and how they judge their own level of QM. For this judgement we will use a multiple choice question with the following options: 1. insufficiently developed; 2. under development; 3. well developed; and 4. excellently developed, including a cyclic quality management policy.

### Statistical analysis

The scores, mean scores and standard deviation (if no normality: median and range) of domains, sub-domains and the overall mean will be computed for both DCGs and DOCs. Ranges of the overall score will be determined to show the differences in the level of QM within DOCs and DCGs.

After one year, dependent t-tests (if no normality: Wilcoxon matched pairs signed rank sum T test) will show whether changes occurred in the overall score and the domain scores. Linear regression analysis will be used to analyse the association between the hours spent on the intervention and the one-year change in overall score.

Univariable regression will be used to assess the relationship between the use of the toolbox, hours spent by the quality advisors and hours the organisation additionally spent on QI (determinants) and change in overall QM (dependent variable). To test generalisability, independent t-tests will be used to check whether there is a difference in the number of patients treated by responding and non-responding organisations. Besides, the median score of the level of QM in the non-responder group will be computed and compared with the level in the responder group.

To check validity of the questionnaire, actual agreement between two respondents of the same organisation will be computed. Since both questionnaires contain a wide variety of questions with three to seven answering categories and on top of that multiple answering was possible, there will be no correction for expected agreement.

All parameters will be tested for normality and the assumptions for regression analysis will be checked. For all tests P-values <0.05 will be considered significant. Analysis will be performed using the SPSS 20.0 statistic software package.

## Discussion

This study will provide an overview of the current level of quality management in diabetes care groups and diabetes outpatient clinics across the Netherlands. It will give insight into the possibility to change this level with a relatively simple intervention. If participating organisations are able to improve QM by our intervention, we expect that this might lead to improved quality of care and subsequently to improved patient outcomes. Tricco et al. already stated that targeting the system of chronic disease management should be regarded important in improving diabetes management [17].

There were no questionnaires available to measure QM in DCGs and DOCs. Therefore, we had to develop these ourselves. This allowed us to operationalise QM specially tailored to QM in DCGs and DOCs. This operationalisation might also be relevant for ACOs and CCGs and other DOCs because they will probably be confronted with the same aspects of QM as well. Therefore, our operationalisation of QM may be an asset for measuring QM at institutional level. The online questionnaires contain multiple response questions which are easy to access and simple to fill out.

Our questionnaire is not completely validated. However, we expect there is sufficient internal validity because of the search strategy and the expert consultation. Specifically, face validity [29] is addressed as draft questionnaires were piloted by experts and their comments were processed; both final questionnaires were weighted by expert panels. Further, the percentage of agreement will be checked by inviting two persons of the same organisations to fill out the questionnaire.

Another limitation we have to address is the bias that might be introduced because of social desirable answering [30]. To overcome this problem, we operationalised management strategies and leadership as much as possible by questioning ‘management strategies’. Social desirability however seems unlikely, because individual feedback was guaranteed in order to enable participants to learn from the results and to stimulate them to improve the QM of their organisation.

Regarding generalisability of the results, it might be possible that especially those organisations will respond, who already have a good level of QM, or, on the contrary, organisations that have only just started their QM. To control for this possible bias, we asked non-responders in one multiple-choice question how they would describe the current level of their QM.

This study aims to attribute to better understanding of the comprehensive elements needed for good diabetes quality management in DCGs and DOCs.

## Abbreviations

T2DM: Patient with type 2 diabetes mellitus; DCGs: Diabetes care groups; DOCs: Diabetes outpatient clinics; ACO: Accountable Care Organization; QM: Quality management; QI: Quality improvement; GP: General Practitioner.

## Competing interests

All authors declare no competing interests.

## Authors’ contributions

GR, CB and JG were responsible for identifying the research question, the design of the study, the acquisition of funding. MC coordinated the study. GR, LL, JG, KV, KG and MC developed the questionnaires. KV designed the website. JG and KV supported the intervention. MC researched the data, conducted the analysis and wrote the manuscript. GR, CB and LL helped to draft the manuscript. All authors read and approved the final manuscript.

## Pre-publication history

The pre-publication history for this paper can be accessed here:

http://www.biomedcentral.com/1472-6963/13/129/prepub

## Supplementary Material

Additional file 1 Search strategy QM questionnaires. Click here for file

## References

[B1] Danaei G Finucane MM Lu Y Singh GM Cowan MJ Paciorek CJ Lin JK Farzadfar F Khang YH Stevens GA National, regional, and global trends in fasting plasma glucose and diabetes prevalence since 1980: systematic analysis of health examination surveys and epidemiological studies with 370 country-years and 2.7 million participants Lancet 2011 378 31 40 10.1016/S0140-6736(11)60679-X 21705069

[B2] Plochg T Klazinga NS Community-based integrated care: myth or must? Int J Qual Health Care 2002 14 91 101 10.1093/oxfordjournals.intqhc.a002606 11954688

[B3] Bodenheimer T Coordinating care–a perilous journey through the health care system N Engl J Med 2008 358 1064 1071 10.1056/NEJMhpr0706165 18322289

[B4] Sluijs EM Wagner C Progress in the implementation of Quality Management in Dutch health care: 1995–2000 Int J Qual Health Care 2003 15 223 234 10.1093/intqhc/mzg033 12803350

[B5] Bohmer RM Lee TH The shifting mission of health care delivery organizations N Engl J Med 2009 361 551 553 10.1056/NEJMp0903406 19657119

[B6] Sluiter AC Van Wijland JJ Arntzenius AB Bots AFE Dijkhorst-Oei LT Van der Does FEE Palmen JVH Potter van Loon BJ Schaper NC Van Balen JAM Landelijke Transmurale Afspraak Diabetes mellitus type 2 [Dutch transmural agreement on Diabetes mellitus type 2] Huisarts Wet 2012 55 1 12

[B7] Struijs JN Mohnen SM Molema CCM de Jong-van Til JT Baan CA Effect van Integrale Bekostiging op Curatieve Zorgkosten. Een analyse voor diabeteszorg en vasculair risicomangement op basis van registratiebestanden Vektis, 2007–2010 [Effect of bundled payment on curative cost of care. An analysis on diabetes care and cardiovascular risk management based on the Vektis database, 2007–2010] 2012 Bilthoven: RIVM

[B8] Struijs JN Baan CA Integrating care through bundled payments–lessons from The Netherlands N Engl J Med 2011 364 990 991 10.1056/NEJMp1011849 21410368

[B9] Luft HS Becoming accountable-opportunities and obstacles for ACOs N Engl J Med 2010 363 1389 1391 10.1056/NEJMp1009380 20925539

[B10] Rosenthal MB Cutler DM Feder J The ACO rules–striking the balance between participation and transformative potential N Engl J Med 2011 365 e6 10.1056/NEJMp1106012 21751898

[B11] DiabeteszorgBeter (Better Diabetes Care) http://www.giantt.nl/DiabeteszorgBeter.pdf

[B12] de Jong-van Til JT Lemmens LC Baan CA Struijs JN De organisatie van zorggroepen anno 2011. Huidige stand van zaken en de ontwikkelingen in de afgelopen jaren [The organisation of care groups in 2011. Current state and recent developments] 2012 Bilthoven: RIVM

[B13] De Grauw WJC NDF Care Standard. Transparency and quality of diabetes care for people with type 2 diabetes 2007 Amersfoort: Nederlandse Diabetes Federatie

[B14] NIV guidelines (guidelines for Dutch endocrinologists) http://www.internisten.nl/gzi2

[B15] van Til JT de Wildt JE Struijs JN De organisatie van zorggroepen anno 2010; Huidige stand van zaken en de ontwikkelingen in de afgelopen jaren [The organisation of care groups in 2010. Current state and recent developments] 2010 Bilthoven: RIVM

[B16] Duckers M Makai P Vos L Groenewegen P Wagner C Longitudinal analysis on the development of hospital quality management systems in the Netherlands Int J Qual Health Care 2009 21 330 340 10.1093/intqhc/mzp031 19689988

[B17] Tricco AC Ivers NM Grimshaw JM Moher D Turner L Galipeau J Halperin I Vachon B Ramsay T Manns B Effectiveness of quality improvement strategies on the management of diabetes: a systematic review and meta-analysis Lancet 2012 379 2252 2261 10.1016/S0140-6736(12)60480-2 22683130

[B18] van Thiel G Oosterwegel M Vermaas A Good scientists make good science 2011 Utrecht: UMC Utrecht

[B19] Care Institutions Quality Act http://wetten.overheid.nl/BWBR0018906

[B20] Wagner EH Austin BT Davis C Hindmarsh M Schaefer J Bonomi A Improving chronic illness care: translating evidence into action Health Aff (Millwood) 2001 20 64 78 10.1377/hlthaff.20.6.64 11816692

[B21] Onderbouwing HKZ-model versie 2008 [Basis of HKZ model, version 2008] http://www.hkz.nl/images/stories/publicaties/def%20HKZ%20Onderbouwing%20HKZ%20model.pdf

[B22] Hertz HS Reimann CW Bostwick MC The Malcolm Baldrige National Quality Award concept: could it help stimulate or accelerate health care quality improvement? Qual Manag Health Care 1994 2 63 72 10137609

[B23] EFQM http://www.efqm.org

[B24] INK-management model http://www.ink.nl/nl/p4bd80e5bc3a81/ink-filosofie.html

[B25] Nabitz U Klazinga N Walburg J The EFQM excellence model: European and Dutch experiences with the EFQM approach in health care. European Foundation for Quality Management Int J Qual Health Care 2000 12 191 201 10.1093/intqhc/12.3.191 10894190

[B26] Wagner C De Bakker DH Groenewegen PP A measuring instrument for evaluation of quality systems Int J Qual Health Care 1999 11 119 130 10.1093/intqhc/11.2.119 10442842

[B27] Standard ENQual hospital pilot questionnaire English 27 12 07 http://www.docstoc.com/docs/112961266/Standard-ENQual-hospital-pilot-questionnaire-English-27-12-07

[B28] Minkman M Ahaus K Fabbricotti I Nabitz U Huijsman R A quality management model for integrated care: results of a Delphi and Concept Mapping study Int J Qual Health Care 2009 21 66 75 10.1093/intqhc/mzn048 18945745

[B29] Streiner DL Norman GR Health Measurement Scales. A practical guide to their development and use 2008 Oxford: University Press

[B30] Savage J Ethnography and health care BMJ 2000 321 1400 1402 10.1136/bmj.321.7273.1400 11099288 PMC1119117

