# Supplementary material for: Defining and improving quality management in Dutch diabetes care groups and outpatient clinics: design of the study
Source: BMC Health Serv Res. 2013 Apr 5;13:129. doi: 10.1186/1472-6963-13-129 (PMC3623730; doi:10.1186/1472-6963-13-129)
Supplement: Additional file 1 — Search strategy QM questionnaires. [file 1472-6963-13-129-S1.pdf]

Appendix

Terms for the search strategy for questionnaires

We searched **pubmed** with the following terms:

("diabetes mellitus, type 2"[MeSH Terms] OR "T2DM"[Ti] OR "DM2"[Ti] OR "diabetes"[Ti] OR "diabetes mellitus"[Ti] OR "type 2 diabetes"[Ti] OR "diabetes type 2"[Ti] OR "type 2 diabetes mellitus"[Ti] OR "diabetes mellitus type 2"[Ti] OR "diabetes mellitus type ii"[Ti] OR "dm2"[Ti] OR "dm type 2"[Ti] OR "dm type ii"[Ti] OR "t2dm"[Ti] OR "type 2 diabetic"[Ti] OR "non-insulin dependent diabetes"[Ti] OR "non-insulin dependent diabetes mellitus"[Ti] OR niddm"[Ti])

AND ("outpatients"[MeSH Terms] OR "outpatient clinics, hospital"[MeSH Terms] OR "ambulatory care"[MeSH Terms] OR "ambulatory care facilities"[MeSH Terms] OR outpatient OR outpatients OR "Ambulatory care" OR "comprehensive health care/methods"[MeSH Terms] OR "comprehensive health care/organization and administration"[MeSH Terms] OR "patient-centered care"[MeSH Terms] OR “patient centered care” [Text Word] OR "long-term care"[MeSH Terms] OR "integrated care" OR "shared care" OR "coordinated care" OR "disease management" OR "transmural care" OR "comprehensive care" OR "intermediate care" OR “chain care” OR “managed care” OR “quality management” OR “HMO” OR “health maintenance organization” OR “ACO” OR “Accountable Care Organizations”)

AND ("quality control"[MeSH Terms] OR "quality assurance, health care"[MeSH Terms] OR (“pdsa quality improvement”) OR (“pdsa cycle”) OR (“pdca cycle”) OR

(“pdsa quality”) OR ("management quality circles"[MeSH Terms]) OR ("total quality management"[MeSH Terms]) OR ("quality improvement") OR ("continuous quality improvement") OR "managed care" OR "quality improvement" OR "quality management")

AND ("accreditation"[MeSH Terms] OR accreditation [Text Word] OR "certification"[MeSH Terms] OR certification[Text Word] OR ("questionnaires"[MeSH Terms] OR questionnaire[Text Word]) OR "assessments, healthcare quality"[All Fields] OR "assessment, process health care"[All Fields])

We searched **Cinahl** using the following terms:

(MM "Diabetes Mellitus, Non-Insulin-Dependent/CO/DI/DH/DT/ED/NU/PR/TH") AND (MM "Quality Assurance/ED/EV/MT/ST/TD") OR (MM "Quality Circles/ED/EV/MT/PC/ST/TD") OR (MM "Decision Making, Organizational/DE/ED/EV/MT/ST/TD") OR (MM "Quality of Health Care/ED/EV/MT/ST/TD") OR (MM "Quality of Care Research/ED/EV/MT/OG/ST/TU/TD") OR (MM "Quality Patient Care Scale/ED/EV/MT/OG/ST/TU/TD") OR (MH "United States Agency for Healthcare Research and Quality+/ED/EV/MT/ST/TD") AND (MM "Disease Management/ED/EV/MT/ST/TD") OR (MM "Multidisciplinary Care Team/ED/EV/MT/ST/TD") OR (MM "Community Health Nursing/ED/EV/MT/ST/TD") OR (MM "Ambulatory Care/ED/EV/OG/MT/ST") OR (MM "Primary Health Care/ED/EV/MT/ST/TD") OR (MM "Nursing Protocols/ED/EV/MT/ST/TD") OR (MM "Patient Care Plans/ED/EV/MT/ST/TD") OR (MM "Health Care Delivery, Integrated/ED/EV/MT/ST") OR (MM "Health Care Reform/ED/EV/MT/ST/TD") OR (MM "Health Maintenance Organizations/ED/EV/MT/ST/TD")

In **Cochrane** the following MeSH descriptors were used: Diabetes Mellitus, Type 2, AND Quality of Health Care AND (Ambulatory Care OR Disease Management OR Patient-Centered Care OR Primary Health Care OR Comprehensive Health Care)

Embase: ('non insulin dependent diabetes mellitus'/exp OR 'non insulin dependent diabetes mellitus')AND ('outpatients'/exp OR 'outpatients' OR 'outpatient clinics' OR 'ambulatory care'/exp OR 'ambulatory care' OR 'comprehensive health care'/exp OR 'comprehensive health care' OR 'patient-centered care'/exp OR 'patient-centered care' OR 'long-term care'/exp OR 'long-term care' OR 'integrated care' OR 'shared care' OR 'coordinated care' OR 'disease management'/exp OR 'disease management' OR 'transmural care' OR 'comprehensive care' OR 'intermediate care' OR 'chain care' OR 'managed care'/exp OR 'managed care' or 'quality management'/exp OR 'quality management' OR 'hmo'/exp OR 'hmo' OR 'health maintenance organization'/exp OR 'health maintenance organization' OR 'aco' OR 'accountable care organizations'/exp OR 'accountable care organizations' OR 'ppo' OR 'preferred provider organization'/exp OR 'preferred provider organization' OR 'epo' OR 'exclusive provider organization' OR 'cdhc' OR 'consumer driven health care group') AND ('pdsa quality' OR 'pdca cycle' OR 'pdsa cycle' OR 'pdsa quality improvement' OR 'management quality circles'/exp OR 'management quality circles' OR 'total quality management'/exp OR 'total quality management' OR 'quality improvement'/exp OR 'quality improvement' OR 'continuous quality improvement' OR 'deming cycle' OR 'quality management'/exp OR 'quality management').
